# Anesthetic management of a pediatric patient with Dravet syndrome: A case report

**DOI:** 10.1097/MD.0000000000032709

**Published:** 2023-01-27

**Authors:** Yuri Hase, Shigeru Takuma, Takayuki Hojo, Yukie Nitta, Nobuhito Kamekura

**Affiliations:** a Department of Dental Anesthesiology, Faculty of Dental Medicine and Graduate School of Dental Medicine, Hokkaido University, Sapporo, Hokkaido, Japan

**Keywords:** Dravet syndrome, epilepsy, general anesthesia, seizure

## Abstract

Dravet syndrome (DS) is a rare and severe myoclonic epilepsy, with onset commonly occurring in infancy. Seizures are triggered by various causes, including fever, bathing, and light stimulus. DS is refractory to drug treatment. Moreover, status epilepticus (SE) can cause serious encephalopathy and epilepsy-related deaths. There are very few reports of general anesthesia in DS patients. Herein, we report our experience with the anesthetic management of a pediatric patient with DS.

**Patient concerns and diagnoses:**

A 5-year-old boy (height, 112 cm; weight, 19 kg) was diagnosed with DS through *SCN1A* genetic testing, which revealed a de novo novel missense mutation. His medical history included drug-resistant epilepsy, developmental delay, and hypotonia. His seizures tended to be triggered daily by a rise in body temperature (BT), bathing, and light stimulus. He could not receive adequate dental treatment due to DS, although he had previously undergone dental treatment under restraint at the pediatric dentistry department of our hospital.

**Interventions and outcomes:**

The patient was scheduled for intensive dental treatment under general anesthesia due to noncooperation, and DS-related limitations. By considering the risk posed by elevated BT, seizure-inducing drugs were avoided, and general anesthesia was completed as planned, uneventfully. Although fluctuation of BT occurred during the procedure, it was finally controlled at the end of anesthesia at about the same level as at anesthesia induction. However, small seizures and a single generalized convulsion were observed accompanied by fever on postoperative day 1. The patient was discharged from the hospital without major problems on postoperative day 3, because of detailed planning and close preoperative cooperation with the attending pediatrician.

**Conclusion:**

It is essential to pay attention to managing BT and to avoid drugs that induce seizures during anesthesia for patients with DS. Cautious preoperative planning for anesthesia based on evaluation of the patient and rapid postoperative response in collaboration with the attending pediatrician is necessary in case an epileptic seizure occurs.

## 1. Introduction

Dravet syndrome (DS) is a rare and severe, drug-resistant myoclonic epilepsy with onset in infancy. It is characterized by seizures triggered by fever and various other causes. DS is associated with sudden unexpected death in epilepsy (SUDEP) and acute encephalopathy with SE.

Reports of general anesthesia in DS are extremely rare. Herein, we describe our experience of general anesthesia procedure for dental treatment in a pediatric patient with DS.

## 2. Case presentation

The patient was a 5-year-old boy (height, 112 cm; weight, 19 kg) who was scheduled for dental treatment under general anesthesia. Although he had undergone dental treatment under restraint at the pediatric dentistry department of our hospital in the past, he could not obtain adequate dental treatment due to DS related restrictions and noncooperation.

His medical history included drug-resistant epilepsy, developmental delay, and hypotonia. He was initially treated by a local doctor upon his first convulsive SE, affecting the right half of his body, at the age of 2 months, and was transferred to our hospital at the next SE 2 months later. Epilepsy was not well-controlled with levetiracetam and valproate and focal epilepsy was suspected. At the age of 7 months, administering topiramate (TPM) reduced the seizures; at 8 months old, he was discharged after additional treatment with lamotrigine, which reduced epilepsy severity. During SE at home, he was treated with oromucosal solution midazolam (MDZ) administered nasally. From the age of 9 months, he repeatedly experienced SE during fever, accompanied by myoclonus. At the age of 1 year, he underwent *SCN1A* genetic testing, which revealed a de novo novel missense mutation, leading to the diagnosis of DS.

He had experienced petit mal seizure for 10 to 20 seconds every day, and grand mal for several minutes about once a week, including various types of seizures, such as focal impaired awareness seizures and myoclonus. Due to continuing focal impaired awareness seizures after 40 °C fever once a month, he was treated preventatively with diazepam suppositories whenever he developed fever. The SE frequency decreased annually. He was treated with MDZ nasally as rescue medication during uncontrolled SE, along with suppositories of diazepam. His seizures tended to be triggered daily by a rise in body temperature (BT), bathing, and light stimulus. As he had intellectual disability, he could not speak and communicate. He had no abnormalities in the heart, respiratory organs, or other vital organs. Moreover, he had hypotonia which had been noted in infancy. He could walk 10 steps on his own at the age of 4 years. At preoperative consultation, he was carried in a stroller because he walked unstably at home. He had no medical history of aspiration pneumonitis. At the time of consultation, he was medicated with sodium bromide, valproate, TPM, stiripentol, clobazam, pranlukast hydrate, L-carbocisteine, and macrogol 4000. Blood examinations and electrocardiogram were unremarkable.

Preoperatively, we consulted with the patient’s attending pediatrician about his condition, how to respond to all types of seizures of the patient, to obtain instructions regarding oral medication, etc. One day before treatment, he was hospitalized along with his mother. Considering that a rise in BT and light stimuli triggered seizures, a private room was reserved in the ward, which allowed control of low room temperature by means of an air conditioner, and he was advised to bed-bath only and to avoid bathing. Moreover, we ensured that the brightness of the wardroom as well as of the dental surgery room was not problematic. On the morning of the day of treatment, all oral antiepileptic drugs were administered as usual.

**Figure 1.**
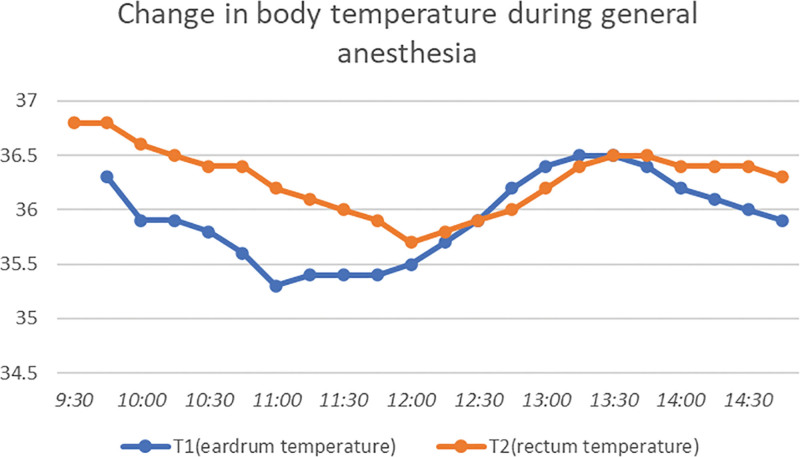
The rectal and eardrum temperature varied as shown without extreme increases or decreases during the operation.

After the patient entered the dental surgery room accompanied by his mother, general anesthesia was induced slowly with sevoflurane 0 to 3% in oxygen at 6 L/min under restraint. After venipuncture and obtaining the control value by neuromuscular monitoring (TOF-watch®, Organon, Ireland), rocuronium 15 mg and a continuous infusion of remifentanil 3.6 mL/h were administered. After nasotracheal intubation was completed without any difficulty, a bispectral index (BIS) electrode was applied and measurement of rectal and tympanic temperatures was started simultaneously. The baseline rectal temperature was 36.8 °C. At the same time, sevoflurane was switched to desflurane 4%. Anesthesia was maintained with air, oxygen, and desflurane, and a continuous infusion of remifentanil. In addition, 1.8 mL of lidocaine 2% with epinephrine 1:80 000 was administered for intraoral infiltration anesthesia during the operation. Acetaminophen (280 mg) was injected intravenously for postoperative analgesia. There were no respiratory or circulatory problems during the operation. Blood pressure varied in the range of 81 to 98/35 to 52 mm Hg; heart rate varied between 67 and 88 bpm; oxygen saturation (SpO_2_) remained at 99 to 100%, and the BIS value ranged stably from 40 to 50. To prevent elevation of BT and maintain the BT as close to that at anesthesia induction as possible, BT was controlled using air conditioning, a blanket, and warming equipment (Bear hugger®, 3M, MN, USA). The rectal temperature varied within 35.9 to 36.2-36.5 °C, which indicated a decrease in BT in the range of 0.3 to 0.6 °C during the operation (Fig. 1). Ten minutes before the end of the procedure, propofol 20 mg was injected to prevent emergence agitation. By monitoring the TOF-watch®, the ratio of train-of-four (TOF) was shown to be 1.0 at 110 minutes after the administration of rocuronium, and no additional muscle relaxants were administered. Dental treatment was completed uneventfully, as planned. Extubation was performed after confirming that muscle function was fully restored, as indicated by a ratio of TOF of 1.0, after administering sugammadex 40 mg and waiting for adequate spontaneous respiration, reflexes, and muscle recovery. After extubation, body movement was observed; however, no seizure or respiratory problems were observed. After confirming that respiratory and circulatory dynamics were stable, the patient was transported to the private room in the ward as planned. The operative time was 4 hours and 54 minutes and anesthesia time was 6 hours and 1 minute. Total infusion volume was 650 mL and urine volume was 30 mL. Three hours after returning to the ward, the patient’s ability to drink water was examined, after which oral antiepileptic drug administration was resumed as usual. postoperatively, BT was 37 °C, and no abnormal temperature elevation was noted, although continuous temperature measurement could not be applied due to noncooperation. However, the next morning at 8:00 am, the patient had fever (BT 38 °C) with gradual rise in temperature accompanied by small seizures and repeated single generalized convulsions. After calling the pediatrician for consultation, the patient was moved to the pediatric department for observation with cooling and administering diazepam and acetaminophen suppositories. Intravenous catheterization was performed and preparation for MDZ infusion was made; however, no medication was administered as the patient did not reach SE. Blood tests performed on postoperative day 1 morning, showed a C-reactive protein level of 2.95 and a white blood cell count of 9900/μL. On postoperative day 3, his BT had decreased, and he was discharged from the hospital.

The parents of the patient provided written informed consent for the publication of this case report

## 3. Discussion

Severe myoclonic epilepsy of infancy was first described by Dravet in France in 1978.^[1]^ The lowest incidence of DS is estimated at 1:45,800 in Japan.^[2]^ This disorder exhibits specific clinical features including onset in the first year of life; temperature-sensitive seizures, which include generalized tonic–clonic and unilateral clonic seizures; drug resistance.^[1]^ Moreover, DS is characterized by high epilepsy-related premature mortality and a conspicuously young mean age at death. Epilepsy-related deaths, most of which result from SUDEP and acute encephalopathy with SE, account for the vast majority of premature mortality in DS (up to 81%).^[3,4]^ High SUDEP rates in DS may be explained by epilepsy severity.^[4]^ Approximately 3-fourths of DS patients have a mutation in the voltage-gated sodium channel gene, *SCN1A*,^[1,2,5]^ which has been suggested as a possible candidate gene related to SUDEP.^[4,6]^

In administering general anesthesia to DS patients, as in our case, it is necessary to eliminate factors that reduce the seizure threshold as much as possible. In particular, one of the main points is the regulation of BT-induced seizures. Additionally, a prompt response in case of epileptic seizure is a requisite.

Childhood fever of DS is the most characteristic and commonly occurring pathology. There are reports of temperature dysregulation in more than 80% of DS patients.^[7]^ In a murine model of DS, impaired thermoregulation^[8]^ and hyperthermia-induced seizure^[9]^ have been demonstrated. In our case, in addition to the difficulty in controlling seizures because of the refractoriness to antiepileptic drug treatment, the patient was receiving TPM, which has side effects such as hypohydrosis, hyperthermia, and a rise in BT, which could had some effects on BT regulation.^[10]^ As expected, perioperative temperature control was challenging. Measures to avoid elevated BT were taken from the time of admission the day before the procedure, as it was a known seizure trigger in our patient. For this reason, room temperature was kept low in a private room of the ward, and instruction was given to avoid bathing on the day before the operation. To maintain BT during anesthesia at the same level as at induction in the operation room, we decreased the room temperature and covered the patient with only a thin blanket. When the BT decreased below 36 °C, the room temperature was raised by several degrees and warming equipment was used at 38 °C for a brief period. Once the patient’s temperature had increased, BT was again controlled by layering additional blankets. At emergence, we were prepared to administer warming in case the patient shivered due to the lengthy operation, but no shivering occurred. Thus, although BT fluctuated during the operation, it was well-controlled at the end of anesthesia to virtually the same temperature as at induction.

In addition to the impaired thermoregulation associated with DS, it is difficult to manage BT during operation under general anesthesia, in which the hypothalamus is suppressed for a long duration. It is frequently mentioned that bacteremia, tissue trauma, dehydration, pulmonary atelectasis, drugs, such as atropine, and environmental factors, such as room temperature and the draping of the patient during surgery, are factors that might be responsible for postoperative fever in children. The incidence of fever after pediatric dental general anesthesia ranged from 20 to 50% in previous studies.^[11,12]^ Most fever cases develop within 24 hours postoperatively, significantly decreasing between 24 and 72 hours postoperatively.^[11,12]^ The longer duration of children’s preoperative fasting and their inability to eat postoperatively might promote postoperative dehydration and fever in children. Another report showed that the method of anesthesia, type of surgery, duration of surgery, and duration of anesthesia were risk factors for postoperative fever in children following cleft lip and palate repair surgery.^[13]^ During operation in our case, adequate infusion of fluids was administered to prevent dehydration, and the patient’s IV line was taken out due to noncooperation, after ensuring that he was able to drink water sufficiently. We could have avoided the fever-induced-seizure on the day after the operation if we could perform continuous BT measurement, although this was not possible, or by giving instructions to the ward to take his temperature more frequently. However, one of the reasons for seizure of DS could be infection. In this case, postoperative blood tests could not exclude the possibility of infection.

It is essential to avoid seizure-inducing drugs when selecting drugs to be administered. For a slow induction, sevoflurane, which has low airway irritation qualities, was administered, but not at high concentrations, to avoid inducing seizure. On the other hand, for intraoperative use, desflurane was selected, which has an anticonvulsant effect. Similarly, remifentanil was continuously administered at a low dose, without fentanyl. Appropriate respiratory management was maintained to avoid hypoxemia and hypercarbia, which could decrease the seizure threshold.

Based on a previous report that intraoperative seizure could be perceived by observing electroencephalogram changes on BIS, a continuous electroencephalogram monitoring was performed using BIS in our case. However, we observed no findings suggestive of epileptic seizures, such as fluctuation and sudden increases in BIS value, or any abnormal waveform, including spike-and-wave patterns.^[14]^ Moreover, no obvious delay in muscle relaxation was seen during TOF monitoring, due to hypotonia.

Preoperatively, the patient’s parents were presented with sufficient information about the risk of perioperative fever and seizure and their consent for the procedure was obtained. In addition, by consulting the attending pediatrician and obtaining his cooperation in case of seizures beforehand, it was possible to ask for rapid pediatric medical examination and treatment at the actual seizure. Consequently, the patient’s fever subsided using only the typical initial response to daily seizures, and he was discharged from the hospital, without development of SE and requirement for special drug administration.

On reflection, more consideration should be given to the postoperative management of BT. To the best of our knowledge, anesthetic management of a pediatric patient with DS has not been described in English previously. Further studies are needed to establish safe perioperative management protocols for DS patients.

In conclusion, we administered general anesthesia for dental treatment in a pediatric patient with DS. For anesthetic management, it is important to eliminate factors that negatively affect seizure thresholds to prevent seizures. In particular, it is essential to pay attention to managing BT increase, which typically triggers seizures. Careful preoperative planning for anesthesia and postoperative rapid response in collaboration with the attending pediatrician is necessary in case an epileptic seizure occurs.

## Author contributions

**Conceptualization:** Yukie Nitta.

Methodology: Yuri Hase.

Project administration: Yuri Hase, Shigeru Takuma, Takayuki Hojo.

Writing—original draft: Yuri Hase.

Writing—review and editing: Yuri Hase, Nobuhito Kamekura.
